# The Massachusetts Metaphysical College and the Penal Code of the State of New York

**Published:** 1911-07

**Authors:** 


### The Massachusetts Metaphysical College and the Penal Code of the State of New York

STATE medicine, sanatory science, preventive medicine is the crown jewel of modern civilisation. All countries which take pride in their progress in social service are intimately concerned and interested in state medicine; and this concern is frequently expressed in statutes protecting the public health, and by laws determining the preparedness, the competency of such persons as would engage in the practice of medicine.

President Eliot, at the time head of Harvard University, in making what might well be considered the most effective address of his life, named state medicine as one of the most encouraging, hopeful and inspiring pursuits of the coming century.

It would, therefore, seem that to the state of Massachusetts and with greater particularity to the members of the medical profession, from which should come the inspiration of precept and example, it would be an occasion of interest to have attention called to an open and flagrant defiance and violation on the part of a Massachusetts corporation, of every essential and fundamental principle of state medicine. In this citation we refer to the business of the M. M. College, and its work of granting or selling the degrees of Quimby-Eddyism, C. S., C. S. B., and C. S. D., which degrees, or any of them, seem to prompt the holder to engage in the practice of medicine, which practice by such person, in the state of New York and many other of the states of America, is unlawful and exposes the offender to arrest, and upon conviction to fine or imprisonment, or to both of such penalties.

The more reliable literature of the subject indicates that the M. M. College was chartered January 31, 1881, and in the first ten years of its existence had some four thousand students.

The extent of the licensing activities of the college is further indicated in the official register of its graduates whose business cards with addresses, office hours, 'phone numbers, the usual information of a business advertisement, appear from month to month in the organ of the college, the *Christian Science Journal.* The number of this journal for May, 1911, devotes some 63 closely printed double columned pages to these advertisements, probably more than three thousand of them, and more than three hundred of them are advertising for business in the state of New York.

The particular interest of the state of New York in the activities of her resident graduates of the M. M. College from her evaluation of state medicine is both general and specific. In general such persons cannot be anything but undesirable citizens, inasmuch as their education at the M. M. College constrains them to hold as a matter of religious belief, and to practise in their daily lives for support, the vicious tenets and Satanic concepts, that all mankind should obstruct, retard, and oppose all public health statutes, laws and ordinances, all state and local boards of health, all provisions for gathering, recording and publishing vital statistics, and all members of the medical profession who believe in or support the same, and that for the very sufficient reasons as taught by the M. M. College that, to these laws, statutes and ordinances of state medicine, to these vital statistics, to these officers of state medicine, and these supporting doctors, the poor, suffering people owe all the morbidity and mortality with which humanity is so greviously afflicted. Even the most casual reader will see that this indictment of state medicine by the M. M. College is, and must be, a most serious matter to any person—(and there are many thousands of such) who holds it to be the truth.

The more specific interest of the state of New York in the undesirable graduates of the M. M. College arises from the fact that every act of these persons in the practice of the art of healing, for which they paid for the teaching of that college, is an unlawful act, in precise and plain violation of the provisions of the laws of the state for the protection of the public health; in fact, is a crime, punishable by fine and imprisonment as was recently set forth by the court in the case of The People of the State of New York against Willis Vernon Cole, defendant, City Magistrate's Court of the City of New York, First Division, Second District. Decided February 11, 1911.

It will now be in order to direct the reader's attention to the suggestion of our title—to a provision of the Penal Code of the State of New York, Section 1530—Public Nuisance Defined. A "public nuisance" consists in unlawfully doing an act which injures or endangers the health or safety of any considerable number of persons; and is a crime against the order and economy of the state.

In our opinion, a corporation is as responsible for its unlawful acts as an individual would be for similar acts, and the M. M. College known to the state of New York through its over three hundred unlawful medical practitioners and their many thousands of faithful followers, all active enemies of state medicine, is plainly a public nuisance and should be so declared by the proper authority, and by the like authority should be effectually abated. The history of the Atlantic School of Osteopathy, recently doing business in this city, and its prompt abatement by our state authorities, is an illustration of how the state of New York protects the public health of our citizens, and also shows what might happen to the M. M. College if that corporation were located within the jurisdiction of our courts.

Might it not be worth the effort for the medical men of Massachusetts to ask themselves the question: Are the problems of state medicine of New York so very different from those of Massachusetts, or is it simply the fact that in New York the laws of public health are intelligently enforced, while in Massachusetts the same are scandalously neglected?

